# Concomitant acute limb ischemia and multiple acute ischemic strokes complicating COVID-19: a case report

**DOI:** 10.11604/pamj.2021.38.275.28712

**Published:** 2021-03-17

**Authors:** Falmata Laouan Brem, Taha Abu-Al-Tayef, Hammam Rasras, Zainab El Mir, Omar El Mahi, Noha El Ouafi

**Affiliations:** 1 Department of Cardiology, Mohammed VI University Hospital, Faculty of Medicine and Pharmacy of Oujda, Oujda, Morocco,; 2 Department of Vascular Surgery, Mohammed VI University Hospital, Oujda, Morocco,; 3 Department of Cardiology, Mohammed VI University Hospital, Epidemiological Laboratory of Clinical Research and Public Health, Oujda, Morocco

**Keywords:** COVID-19, arterial thrombosis, acute ischemic stroke, anticoagulation, case report

## Abstract

Since the spread of the coronavirus disease 2019 (COVID-19) pandemic, cardiovascular complications are interestingly increasing, particularly thrombotic events, especially in those requiring intensive care. Venous thromboembolism is well known to occur in patients infected by the SARS-CoV-2, but only a few arterial thromboembolism cases have been previously reported. Herein, we report the case of a COVID-19 complicated by a concomitant acute right limb ischemia and multiple acute ischemic strokes. This rare case emphasizes the hypercoagulable state described in COVID-19 patients and the need for anticoagulation therapy to prevent these severe complications.

## Introduction

Since the spread of the novel coronavirus (COVID-19) pandemic, thrombotic disorders are widely reported in patients with COVID-19. Although blood clots pathogenesis remains unclear, endothelial cell dysfunction, the elevation of circulating prothrombotic blood factors (fibrinogen, D-dimers) are mechanisms that several studies have proposed to explain the hypercoagulable state during the COVID-19 disease [1,2]. Besides, this hypercoagulable seems to induce both venous and arterial thrombosis [3]. Nevertheless, only a few arterial thromboembolism cases have been previously reported. Herein, we report the case of a COVID-19 complicated by a concomitant acute right limb ischemia and multiple acute ischemic strokes.

## Patient and observation

**Figure 1:**
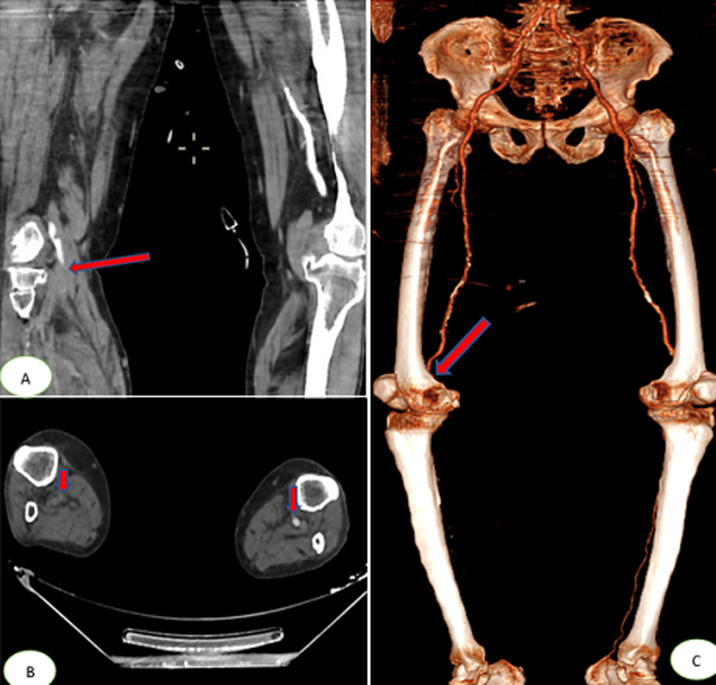
(A,B,C) computed tomography angiography of the lower limbs showing an abrupt arrest in the right popliteal artery (red arrows)

**Figure 2:**
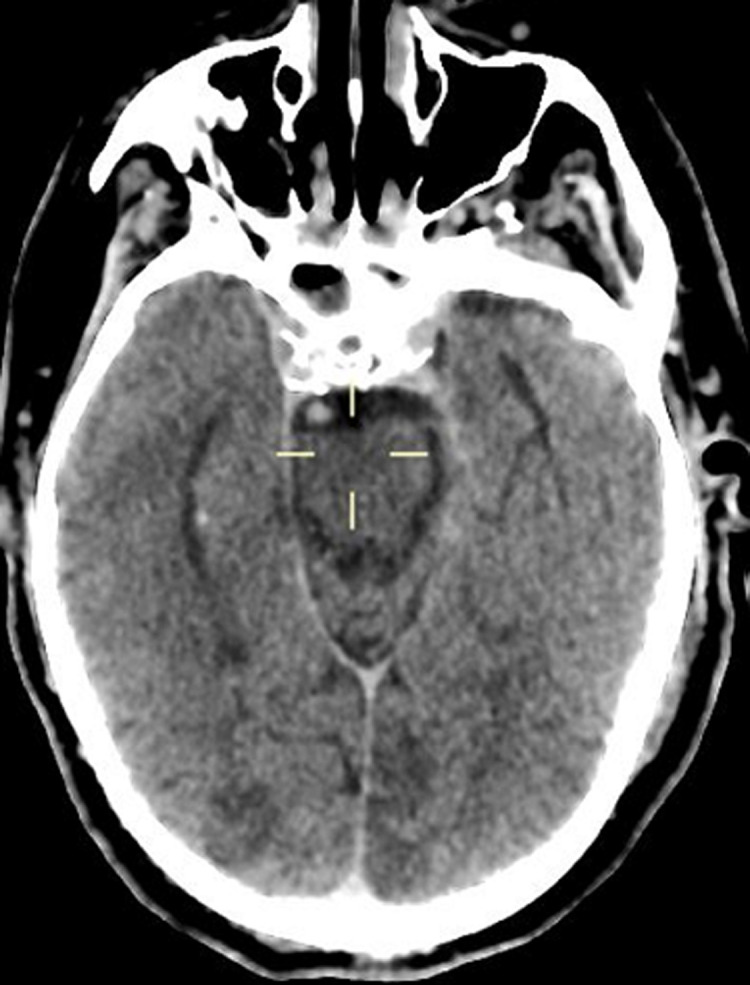
cerebral CT scan revealing multiple hypodense areas cortico-subcortical bilateral parietal and occipital related to multiple strokes

**Figure 3:**
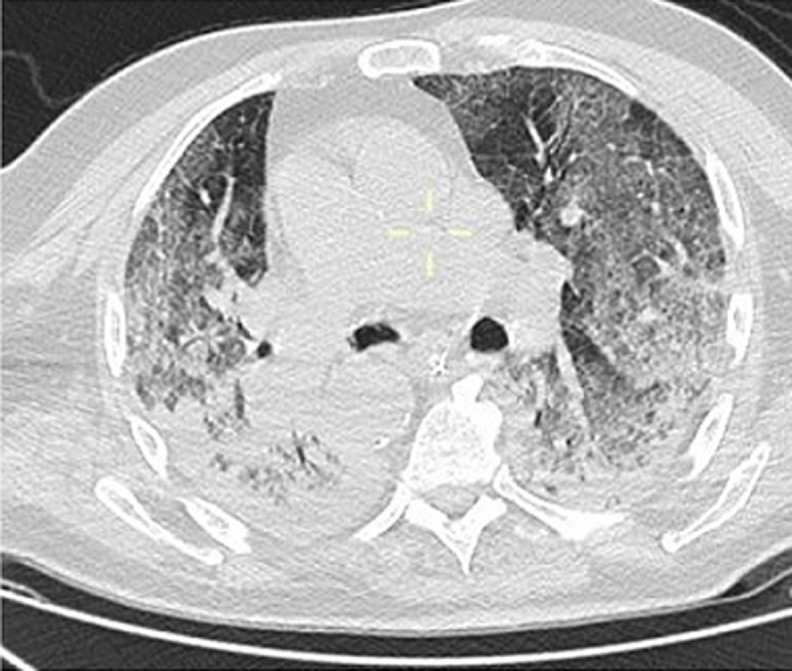
chest CT scan showing suggestive radiological finding related to COVID-19-pneumonia at the lung parenchyma including peripheral distribution of ground-glass opacities associated with consolidation

A 65-year-old man with hypertension and diabetes presented to the emergency department after six days of fever, cough, diarrhea and, headache with a previous history of unprotected contact with his brother, who tested positive for COVID-19 infection. He was stable hemodynamically oxygen saturation at 95% in room air. The SARS-CoV-2 RT-PCR performed on nasopharyngeal swabs confirmed the diagnosis of COVID-19. The patient was hospitalized in general wards and received hydroxychloroquine 400mg twice a day with a prophylactic dose of anticoagulation therapy (enoxaparin). Six days after, he deteriorated with increased breathing, oxygen saturation at 65% with 15L/min; bradycardia with heart rates at 40, and required intubation with mechanical ventilation for COVID-19-related acute respiratory distress syndrome (ARDS). He continues receiving in addition to hydroxychloroquine and empiric therapeutic anticoagulation therapy, ceftriaxone 2g once a day and antiviral (lopinavir 400mg). Four days later, the physical examination revealed a right lower limb mottled, cold, with the absence of popliteal, posterior tibial and foot pulses. An urgent venous and arterial Doppler ultrasound was performed which showed no vascular flow at the right popliteal artery level. Urgent lower limbs computed tomography angiography (CTA) showed an abrupt arrest in the right popliteal artery Figure 1 (A,B,C). A cerebral computed tomography (CT) scan was also performed, which showed multiple hypodense areas cortico-subcortical bilateral parietal and occipital related to multiple strokes (Figure 2). Chest CT scan revealed suggestive radiological findings of COVID-19 pneumonia. Visual score extension: 75% (Figure 3).

Laboratory findings showed elevated white blood cells (WBC) (27680 elements/mm^3^); lymphopenia (400 elements/mm^3^); elevated levels of inflammatory markers (CRP at 300mg/L, a high level of ferritin at 1642.16 ng/mL, a high fibrinogen level at 5.1g/L), high D-Dimer level at (32300ng/mL), decreased platelets levels (80000 elements/mm^3^), acute kidney failure with a creatinin level at 26.27mg/l (creatinin clearance MDRD 26.18ml/min), and elevated high-sensitivity troponin level at 1150,2ng/L. Our center's vascular surgery department was consulted, and they declared the limb unsalvageable and advised for the right limb above the knee amputation which was performed successfully. Therapeutic anticoagulation with Tinzaparin 14000UI per day was introduced to prevent any other thrombotic events. Given the reported prevalence of antiphospholipid antibodies in COVID-19 patients who developed strokes [4] and also given the occurrence of both acute limb ischemia and ischemic stroke, we performed a thrombophilia study including protein S, protein C, antithrombin III and antiphospholipid antibodies which showed a normal result. He further deteriorated with end-stage renal failure (creatinine 56 mg/L, MDRD: 10.9ml/min) and hyperkalemia at 7mmol/L, requiring continuous hemofiltration. Sadly, he passed away 2 days later.

## Discussion

The hypercoagulable state described in patients infected by the SARS-CoV-2 seems to induce both venous and arterial thrombosis. Nevertheless, the incidence of arterial thrombosis among patients with COVID-19 is not clearly defined. A recent review study on five cohorts of 90 critical ill COVID-19 patients reported a pooled incidence of 4.4% of arterial thrombotic events, and most of them were located in the limb arteries (39%) [5]. We reported a COVID-19 patient who presented acute limb ischemia and multiple acute ischemic strokes while in a coma. There are only a few reports of arterial thrombosis leading to ischemic stroke and acute limb ischemia. In fact, just a few acute arterial thrombosis cases in the mesenteric, aorta, limb and cerebral arteries were reported [6-9]. The incidence of strokes reported in patients infected by the SARS-CoV-2, range from 0.9%-2.7% [10]. The hypercoagulable state during the COVID-19 leading to thrombotic disorders could explain why during the COVID-19 infection, most strokes that occurred seem to be related to large vessel occlusion (LVO) [11]. Nevertheless, it's difficult to say if the SARS-CoV-2 infection caused the stroke or whether the stroke would have occurred regardless of the infection. Several studies were conducted to assess this relationship between strokes and COVID-19.

Cerasti *et al*. [12] presented the case of a COVID-19 who developed multiple acute ischemic strokes. COVID-19 disease tends to be linked to an elevated thrombotic condition, which can be associated with large artery thrombosis. Furthermore, a case series demonstrated that hospitalized patients treated for stroke due to LVO, more than half tested positive for COVID-19 [13]. Besides, a recent study reported that cryptogenic strokes were twice more prevalent in patients who tested positive for COVID-19 than both those who were tested negative and a control group consisting of patients treated in the same period in 2019 [14]. Although our patient was negative for antiphospholipid antibodies testing, current data reported a high prevalence of antiphospholipid antibodies in seven patients with brain-infarct (83.3 vs. 26.9%, P <0.05) [4] Zhang *et al*. presented the case of a COVID-19-patient with multiple cerebral and limb infarcts in the presence of antiphospholipid antibodies [15]. Antiphospholipid antibodies have been linked to various viruses, including Hepatitis C and HIV. However, it is unclear whether their involvement in the SARS-CoV-2 infection may predispose to relevant coagulopathy.

The mortality in COVID-19-patients with arterial thrombosis and amputation´s rate in those with a lower extremity arterial thrombosis are higher than in those without COVID-19 [16]. A recent study carried out on 1419 patients reported 1% of patients developing arterial thrombotic events, including acute ischemic strokes and acute lower limb ischemia with a mortality rate of 28.6%, and they seem to occur in those with a severe ill [17]. Anticoagulation therapy in COVID-19-patients has been shown to decrease mortality [18]. However, just a few studies have demonstrated favorable clinical results from the use of antiplatelet therapy for arterial thromboprophylaxis in COVID-19 patients [19].

## Conclusion

The exact pathogenesis of COVID-19 infection leading to arterial thrombosis, especially strokes, is not yet understood. However, this rare case emphasizes the hypercoagulable state described in COVID-19-patients leading to a novel and uncommon form of thrombotic events and the need for anticoagulation therapy to prevent these serious complications. Physicians should maintain a high clinical suspicion index to identify patients at a high VTE-risk to prevent and diagnose these thrombotic events.
